# Accuracy of musculoskeletal imaging for the diagnosis of polymyalgia rheumatica: systematic review

**DOI:** 10.1136/rmdopen-2015-000100

**Published:** 2015-08-13

**Authors:** Sarah Louise Mackie, Gouri Koduri, Catherine L Hill, Richard J Wakefield, Andrew Hutchings, Clement Loy, Bhaskar Dasgupta, Jeremy C Wyatt

**Affiliations:** 1 Leeds Institute of Rheumatic and Musculoskeletal Medicine, University of Leeds, Leeds, UK and NIHR Leeds Musculoskeletal Biomedical Research Unit, UK; 2 Leeds Teaching Hospitals NHS Trust, Leeds, UK; 3 Harrogate and District NHS Foundation Trust, Harrogate, UK; 4 York Teaching Hospital NHS Foundation Trust, UK; 5 University of Adelaide, The Queen Elizabeth Hospital, Adelaide, Australia; 6 Department of Health Services Research and Policy, London School of Hygiene and Tropical Medicine, London, UK; 7 University of Sydney, Sydney, Australia; 8 Huntington Disease Service, Westmead Hospital, Sydney, Australia; 9 Southend University Hospitals NHS Trust, UK; 10 Leeds Institute of Health Sciences, University of Leeds, UK

**Keywords:** Polymyalgia Rheumatica, Ultrasonography, Magnetic Resonance Imaging

## Abstract

**Objectives:**

To review the evidence for accuracy of imaging for diagnosis of polymyalgia rheumatica (PMR).

**Methods:**

Searches included MEDLINE, EMBASE and PubMed. Evaluations of diagnostic accuracy of imaging tests for PMR were eligible, excluding reports with <10 PMR cases. Two authors independently extracted study data and three authors assessed methodological quality using modified QUADAS-2 criteria.

**Results:**

26 studies of 2370 patients were evaluated: 10 ultrasound scanning studies; 6 MRI studies; 1 USS and MRI study; 7 18-fluorodeoxyglucose-positron emission tomography (PET) studies; 1 plain radiography and 1 technetium scintigraphy study. In four ultrasound studies, subacromial-subdeltoid bursitis had sensitivity 80% (95% CI 55% to 93%) and specificity 68% (95% CI 60% to 75%), whereas bilateral subacromial-subdeltoid bursitis had sensitivity 66% (95% CI 43% to 87%) and specificity 89% (95% CI 66% to 97%). Sensitivity for ultrasound detection of trochanteric bursitis ranged from 21% to 100%. In four ultrasound studies reporting both subacromial-subdeltoid bursitis and glenohumeral synovitis, detection of subacromial-subdeltoid bursitis was more accurate than that of glenohumeral synovitis (p=0.004). MRI and PET/CT revealed additional areas of inflammation in the spine and pelvis, including focal areas between the vertebrae and anterior to the hip joint, but the number of controls with inflammatory disease was inadequate for precise specificity estimates.

**Conclusions:**

Subacromial-subdeltoid bursitis appears to be the most helpful ultrasound feature for PMR diagnosis, but interpretation is limited by study heterogeneity and methodological issues, including variability in blinding and potential bias due to case–control study designs. Recent MRI and PET/CT case–control studies, with blinded readers, yielded promising data requiring validation within a diagnostic cohort study.

**Key messages**

**What is already known about this subject?**

- Provisional American College of Rheumatology/the European League Against Rheumatism (ACR/EULAR) classification criteria for polymyalgia rheumatica (PMR) incorporate several optional ultrasound features, with intra-articular and extra-articular features of inflammation weighted equally.

**What does this study add?**

- Subacromial-subdeltoid bursitis is significantly more discriminatory for PMR compared to glenohumeral synovitis, in four studies with ultrasound data on both features.
- Data mostly come from diagnostic case-control study designs, which can overestimate values for sensitivity and specificity.

**How might this impact on clinical practice?**

- When evaluating patients with suspected PMR, clinicians may consider extra-articular locations of inflammation such as bursitis as supportive, but must bear in mind that there may be biases in current estimates of sensitivity and specificity of these findings.

Polymyalgia rheumatica (PMR) is an age-associated, inflammatory musculoskeletal disease with a lifetime risk of 2.4% for women and 1.7% for men,^1^ and affects 0.7% of the population over the age of 50 years.^2^ Patients report pain and stiffness of the shoulder and/or hip girdles, usually with elevation of inflammatory markers such as C reactive protein and erythrocyte sedimentation rate.^3^ Accurate diagnosis of PMR is essential, given the impact of PMR on quality of life unless it is treated with systemic glucocorticoids, usually for a year or more.^4^ Long-term glucocorticoids produce a significant risk of adverse events.^5–8^ However, PMR can be mimicked by many other conditions,^9^ many of which also respond initially to glucocorticoids. None of the various sets of classification criteria for PMR has yet been fully validated for clinical diagnostic use. There remains a need for additional tests providing diagnostic information, especially where the diagnosis is not clear-cut.

In PMR, there is inflammation in and around the shoulders and hips;^3^ this can often be visualised using imaging.^10^ Based on small, single-centre studies, it has been hypothesised that PMR compared to RA has predominantly extra-articular rather than intra-articular imaging abnormalities.^11–14^ However, the latest, data-driven provisional international classification criteria for PMR give equal weighting to extra-articular and intra-articular ultrasound features.^15^ Since extra-articular features such as subacromial-subdeltoid bursitis (SAB) and trochanteric bursitis are commonly seen with normal ageing,^16^ ^17^ it is important to compare any imaging findings with those from non-PMR controls of similar ages.

The objective of this study was to review the evidence regarding the accuracy of musculoskeletal imaging for the diagnosis of PMR.

## Methods

### Data sources and searches

The systematic review protocol was uploaded to the PROSPERO database before running searches (registration number CRD42013005734). The reference standard was defined as a rheumatologist's diagnosis of PMR, without any better explanation of the presenting symptoms found during follow-up. Potential sources of heterogeneity, including study setting, eligibility criteria, technical aspects of the imaging and glucocorticoid therapy were pre-defined. A PICO-structured search was conducted to identify relevant studies in Pubmed, Ovid MEDLINE (1966−) and EMBASE (including EMBASE Classic) (table 1).

**Table 1:** Search strategy

Pubmed:	Polymyalg* AND (imaging OR ultrasono* OR sonograph* OR echogr* OR “computed tomography” OR “computer assisted tomography” OR “bone scan” OR “nuclear medicine” OR “scintigraph*” OR “PET” OR “positron” OR “MRI” OR “magnetic”)
Ovid Medline:	
1	Polymyalgia Rheumatica/
2	polymyalgi$.mp
3	PMR.tw
4	exp Rheumatic Diseases/
5	3 and 4
6	1 or 2 or 5
7	human/
8	(editorial or comment or historical article or review).pt
9	7 not 8
10	exp “diagnostic imaging”/
11	(diagnostic imaging).mp
12	ri.fs
13	ra.fs
14	us.fs
15	mri.mp
16	(magnetic resonance).mp
17	(mr imaging).mp
18	mr scan$
19	mr.ti
20	exp ultrasonography/
21	ultrasound.mp
22	ultrason$.mp
23	echograph$.mp
24	sonograph$.mp
25	doppler$.mp
26	us.ti
27	scintigraph$.mp
28	positron.mp
29	PET.ti
30	ct.ti
31	radiograph$.mp
32	x-ray$.mp
33	or/10–32
34	6 and 9 and 33
Ovid EMBASE:	
1	exp rheumatic polymyalgia/
2	polymyalgi$.mp
3	PMR.tw
4	exp rheumatic disease/
5	3 and 4
6	1 or 2 or 5
7	limit 6 to human
8	limit 6 to editorial
9	limit 6 to review
10	7 not (8 or 9)
11	diagnostic imaging.mp.
12	exp diagnostic imaging/
13	radiodiagnosis/
14	exp echography/
15	exp computer assisted tomography/
16	exp nuclear magnetic resonance imaging/
17	exp positron emission tomography/
18	ct.ti
19	(mr imaging).mp
20	(magnetic resonance).mp
21	mri.mp
22	mr.ti
23	pet.mp
24	positron.mp
25	scintigraph$.mp
26	sonograph$.mp
27	ultraso$.mp
28	echograph$.mp
29	doppler$.mp
30	us.ti
31	exp ultrasound
32	di.fs
33	radiograph$.mp
34	x-ray$.mp
35	or/11–34
36	10 and 35

The search was performed by combining the following search terms: polymyalgia/polymyalgic and (ultrasound or radiograph or X-ray or imaging or CT or MRI or PET or CT or isotope bone scan or positron emission tomography or MR). No language restrictions were made, in case the abstract reveals useful information.

### Study selection

A study was eligible if it included humans with either suspected PMR (diagnostic cohort design), or both a PMR group and a comparator non-PMR group (diagnostic case–control design), with systematic application of imaging test(s). Expert (rheumatologist) diagnosis was the minimum acceptable reference standard. Diagnostic accuracy data had to be extractable in 2×2 format (true positives, true negatives, false positives, false negatives). Non-systematic review articles, case reports and case series of less than 10 patients were excluded. No language restrictions were made. Case reports were excluded by the reviewers manually, rather than by using filters. Meeting abstracts (previous 2 years of British Society for Rheumatology (BSR), European League against Rheumatism (EULAR) and American College of Rheumatology (ACR) conferences) were also screened, and experts in the field were contacted, to identify studies potentially in press or not fully published. Citations were exported to EndNote, duplicates removed in EndNote and results exported to Microsoft Excel.

### Data extraction and quality assessment

A study quality assessment tool, based on QUADAS-2,^18^ and encompassing internal validity (risk of bias: test reliability, blinding to index test/clinical information, incorporation bias, diagnostic review bias) and external validity (relevance to our review question: participant selection, spectrum of disease and comparator condition, timing of test in relation to glucocorticoid treatment) was agreed in advance. Two reviewers (SLM and GK) independently extracted study characteristics (design, clinical spectrum, reference standard) and diagnostic accuracy data for the index test(s) of each study. Corresponding authors were contacted by email where queries arose. Assessment of methodological limitations and between-study clinical heterogeneity was guided by the study quality assessment tool. Data were entered into Review Manager V.5.2 (RevMan) and exported to Excel.

### Data synthesis and analysis

For each imaging feature, where 4 or more studies were available, meta-analysis was performed in Stata SE V.12 (StataCorp, Texas, USA) with calculation of overall sensitivity, specificity and likelihood ratios (LRs) using the bivariate model,^19^ and graphed using RevMan, allowing visualisation of between-study statistical heterogeneity. Influential studies were identified by plotting Cook's distance for each study. Where fewer than four studies were available, 95% CIs for sensitivity, specificity and LRs for each study were calculated using a spreadsheet.^20^ If a cell in the 2×2 table for a study contained a 0, 0.5 was added to each cell to avoid division-by-zero.

To directly compare accuracy of specific couples of tests, we used Hierarchial Summary Receiver-Operator Characteristic Curve (HsROC) modelling,^21^ with test type as a covariate. We did so with paired data only (data from studies where both tests were evaluated together), to control for study-based biases. We first assessed whether the sROC's for the two tests had similar shapes (the beta parameter), as sROC's with different shapes will cross and whether one test is better than the other or not becomes threshold-dependent.^22^ Where the two sROC's had similar shapes, we were able to compare overall accuracy using the α parameter (indicating proximity to the top left hand corner of the ROC space). Analysis was performed using PROC NLMIXED in SAS V.9.3 (SAS Institute, North Carolina, USA).

## Results

**Figure 1:**
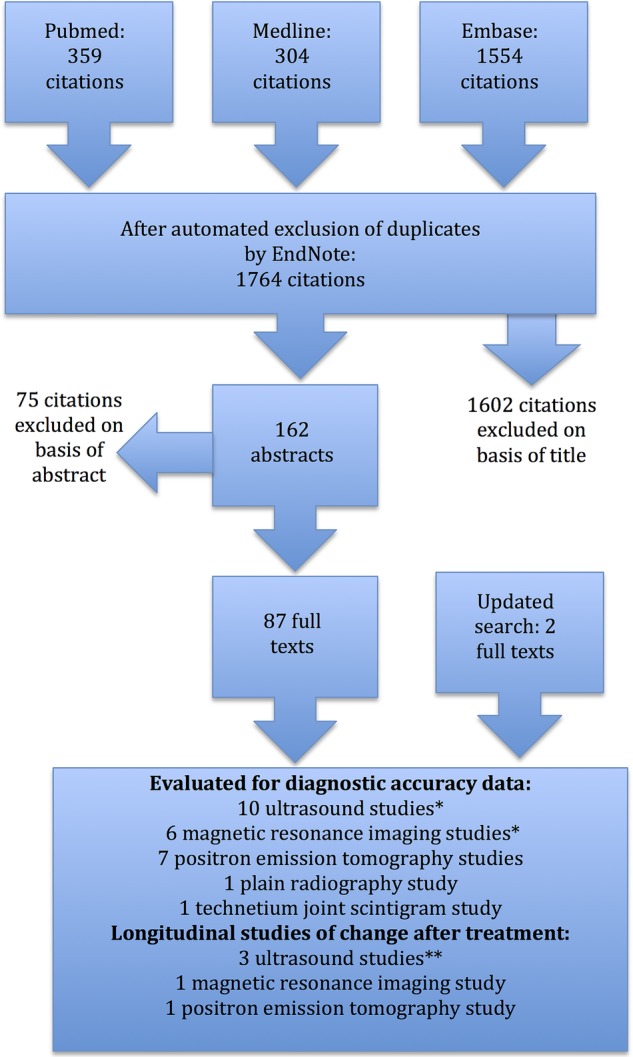
Flow chart for systematic review.

Literature searches were completed on 2 October 2013, yielding 1764 citations (figure 1). We identified 87 articles for full text review of which 23 studies from the original searches were chosen for full evaluation, with three further added on updating searches (January 2015): 10 ultrasound scanning studies (including one published in full text on the updated search^23^); 6 MRI studies; 1 USS and MRI study; 7 18-fluorodeoxyglucose-positron emission tomography (PET) studies (including two published in full text when the search was updated^24^ ^25^); 1 plain radiography^26^ and 1 technetium scintigraphy^27^ study. These last two studies did not meet our review inclusion criteria, one because of a lack of clear distinction between PMR and non-PMR^26^ and the other because it was published in 1976 and we could not exclude the possibility that changes in definition of the diagnostic reference standard may have occurred since then.^27^ Additionally, we reviewed four longitudinal studies.^28–31^ Vascular imaging studies in patients with a diagnosis of PMR were also initially reviewed (six ultrasound and two PET), but subsequently excluded as the primary purpose of these studies was to diagnose giant cell arteritis in patients presenting with PMR symptoms.

Study characteristics and results of quality assessment are shown in table 2. All but one of the studies we identified used a diagnostic case–control design, which is associated with inflation of sensitivity and specificity estimates because of the ‘grey cases’ seen in real-life clinical practice but omitted from the study.^32^ Other common sources of bias in this analysis included incomplete blinding of the person(s) performing the imaging test, diagnostic review bias (incomplete blinding of the diagnostician acting as reference standard) and spectrum bias (studies were generally conducted in academic rheumatology centres) (table 2). Table 3 summarises the accuracy of each imaging feature in PMR, using meta-analysis where appropriate. Original data used to create this table and further details regarding comparator subpopulations are found in the online supplementary. Many different abnormalities were reported by the studies, reflecting the widespread localisation of inflammation in PMR.

**Table 2:** Assessment of methodological quality in diagnostic studies: summary of major biases identified

Study	Index test: imaging modality	Who performed index test, were they blinded to clinical data, was inter/intra-rater reliability reported?	Prospective study?	Does PMR spectrum appear realistic according to information given? Did any also have GCAs?	Consecutive selection of participants?	Comparator condition(s): realistic?	Reference standard; who performed it, when?	Did all participants receive all tests?	Free from incorporation bias?	Free from diagnostic review bias?	Did participants have index test before receiving glucocorticoid treatment?
*Musculoskeletal ultrasound (MSK USS)*											
Dasgupta *et al*^15^*†	MSK USS shoulders, hips	Rheumatologist or radiologist, one per site; reliability reported separately (Scheel *et al*, 2009); at some sites sonographer was clinical assessor	Yes	Yes; none had GCA	No	Yes: >50 years, <12 weeks’ history of bilateral shoulder pain, not felt to be PMR	Clinical diagnosis; by investigator; after 6 months	5 PMR and 15 controls did not have scans	Yes—diagnosis made before USS, and assessors told not to use USS findings in making diagnosis	Sonographer and clinical assessor were sometimes same person	Yes
Ruta *et al*^39^*†	MSK USS shoulders	Single rheumatologist-sonographer blinded to clinical data; reliability not reported	Yes	Maybe: relapsing PMR (new-onset bilateral painful shoulder and prior diagnosis PMR); none had GCA	Yes	Maybe: relapsing RA (new-onset bilateral painful shoulder and prior diagnosis of RA)	PMR: clinical diagnosis+Healey criteria; RA: ACR 2010 criteria; by treating rheumatologist	Yes	Yes	Yes	No; were on ≤10 mg prednisolone; most were on 2–4 mg; treatment did not seem to affect USS findings
Falsetti *et al*^44^†	MSK USS at multiple sites	Single rheumatologist-sonographer, not blinded to clinical data; reliability not reported	Yes	Yes: all participants referred from primary care with polymyalgic syndrome fulfilling Bird criteria; one developed GCA later. All participants drawn from this same population (single-gate study design). 29/61 (47.5%) had final diagnosis PMR. Many of those with RA were seropositive			Clinical diagnosis; by 2 rheumatologists, after 1 year	Yes	No	No	Yes
Cantini *et al*^34^	MSK USS hips and MRI pelvic girdle	Two radiologists for each test (unclear whether these were same people), unclear whether blinded to clinical data (note alternating recruitment of cases/2 controls); reliability not reported	Yes	A subset: PMR with pelvic girdle involvement; 3 also had biopsy-proven GCA; none developed RA (1987 ACR criteria) after average follow-up 26 months	Yes	Maybe: next 2 consecutive outpatients >50 years with active rheumatic disease (RA/PsA/OA) and bilateral hip ache	Clinical diagnosis+Healey criteria PMR, followed up to ensure no evolution to RA	Only 10 of 40 controls had MRI (unclear how these were selected)	Yes	Unclear	Yes for PMR; unclear for controls
Frediani 2002†^45^	MSK USS at multiple sites	Two rheumatologist-sonographers, blinded to diagnosis; “medium rates concordance [agreement]” reported but no test statistics quoted	Yes	Yes: “PMR patients with a relatively certain diagnosis”—Healey criteria; 2 also had GCA	Yes	No: RA (ARA 1987 criteria); SpA (ESSG criteria)	Clinical diagnosis+Healey criteria PMR; 2-year follow-up to confirm diagnosis	Yes	Yes	No, but diagnosis not changed after USS	Yes
Cantini *et al*^33^	MSK USS shoulders	Two radiologists together, blinded to clinical diagnosis (but note recruitment of 2 controls after each case); reliability not reported	Yes	Yes: >1 month pain neck and shoulder girdle; morning stiffness> 1 h; ESR>40; 5 also had biopsy proven GCA; follow-up for mean 8 months to exclude those fulfilling 1987 ARA RA criteria	Yes	Maybe: next 2 consecutive outpatients >50 years with bilateral shoulder aching, stiffness (RA/PsA/SpA/OA/FM/CTD)	Clinical diagnosis+Healey criteria; by 1 of 4 rheumatologists; follow-up to confirm diagnosis	Yes	Yes	Unclear; but participant selection protocol implies participants did not switch between case/control groups	Yes for PMR, unclear for controls
Coari *et al*^38^	MSK USS shoulders	Two rheumatologist-sonographers, unclear whether blinded to clinical data; reliability not reported	Not stated but implied	No: treated PMR; not stated whether any had GCA	Not stated	No: treated; one-third of RA patients erosive	Clinical diagnosis (ARA 1987 for RA); not stated by whom or whether followed up	Only PMR each had both shoulders scanned; unit of analysis was shoulder not patient	Yes	Unclear	No
Lange *et al*^46^	MSK USS shoulders	Not stated; reliability not reported	Not stated but implied	Yes: >60 years, pain and several hours’ morning stiffness of shoulders, neck and/or pelvic girdle, limited motion in neck and shoulder, ESR>45, response to prednisolone 30 mg or less); 6 had headache, 2 had biopsy-proven GCA	Not stated	Maybe: “initially had similar complaints (to the PMR cases) … involvement of arthritis in additional joints and bony erosions”	Clinical diagnosis; not stated by whom or whether followed up	Yes	Yes (implied but not stated)	Unclear	Yes (implied but not stated)
Lange *et al*^47^	MSK USS shoulders	Not stated; reliability not reported	Not stated but implied	Yes: >60 years, pain and several hours’ morning stiffness of shoulders, neck and/or pelvic girdle, >4 weeks duration symptoms, ESR>45, response to prednisolone 30 mg or less); 5 had headache, 4 had biopsy-proven GCA	Not stated	Maybe: “initially had similar complaints (to the PMR cases) … involvement of arthritis in additional joints and bony erosions”	Clinical diagnosis; not stated by whom or whether followed up	Yes	Yes (implied but not stated)	Unclear	Yes (implied but not stated)
Macchioni *et al*^23^	MSK USS shoulders, hips	Single rheumatologist-sonographer; blinding to clinical data not stated; reliability not reported	No	Yes: patients seen with suspected PMR; patients with GCA excluded	Yes	No: patients in early arthritis clinic; no requirement for comparable symptoms	Clinical diagnosis; confirmed at 1 year by 2 lead authors	Yes	Unclear	No	Yes
*MRI*											
Salvarani *et al*^37^	1.5 T MRI lumbar spine (bursitis)	Radiologist; blinded to clinical findings and diagnosis; reliability not reported	Yes	A subset: PMR by Chuang criteria+pelvic girdle symptoms; none had GCA	Yes	Maybe: treated patients with lumbar pain (SpA/OA/RA)	Clinical diagnosis+Chuang criteria, followed up for 10–16 months to exclude RA (ARA 1987) or other conditions	Yes	Yes	Yes	Yes for PMR, unclear for controls
Cimmino *et al*^40^	0.2 T MRI hands (extremity MRI)—tenosynovitis	Two rheumatologists and one PhD, blinded to diagnosis; reliability not reported but Parodi *et al* 2006 quoted in support	Yes	Yes: PMR by Chuang criteria; none had GCA	Yes for PMR, not for controls	No: Healthy controls of similar ages, no mention of symptoms	Clinical diagnosis+Chuang criteria, followed for 8–124 months to exclude GCA, RA and other erosive disease	Yes but 4 hands could not be interpreted	Yes	Yes	Yes
Salvarani *et al*^36^	1 T MRI cervical spine (bursitis)	One radiologist, blinded to clinical data and diagnosis (but note alternating recruitment of cases, controls); reliability not reported	Yes	Yes: PMR (reference Salvarani review 2002); none had GCA	Yes	No: Next patients with neck pain seen after PMR patients	Clinical diagnosis+criteria; followed for 10–16 months to exclude other conditions	Yes	Yes	Yes	Yes
Marzo *et al*^13^	1.5 T MRI of most swollen hand	One assessor per MRI feature, blinded to clinical data; reliability not reported	Yes	No: Bird criteria+MCP joint swelling	Yes for RA, not stated for PMR	No: ARA 1987 criteria+MCP joint swelling	Clinical diagnosis+Bird criteria; followed for mean of 6 years	Yes	Yes	Yes	Yes except for one PMR patient
McGonagle *et al*^12^	1.5 T MRI shoulder	Two radiologists, blinded to clinical data; reliability not reported	Yes	Yes: untreated PMR and bilateral shoulder disease without peripheral arthropathy	No	No: early RA fulfilling 1987 ARA criteria	Clinical diagnosis; no follow-up reported to exclude other conditions	Only 6/14 PMR patients had both shoulders imaged	Yes	Yes	Yes for PMR; not for 8/14 RA
Salvarani *et al*^48^	0.5 T MRI shoulder	One radiologist, blinded to clinical data and diagnosis; reliability not reported	Yes	Yes: Healey criteria PMR; none had GCA	Unclear	No: elderly-onset RA by modified 1987 ARA criteria, with clinical evidence shoulder involvement	Clinical diagnosis+Healey criteria; no follow-up reported to exclude other conditions	The first 4 PMR had both shoulders imaged; after that only one shoulder	Yes	Yes	Yes
*^18^F-fluorodeoxyglucose—positron emission tomography (FDG-PET)*											
Yamashita *et al*^35^	FDG-PET/CT whole body	Not stated who reported test; unclear whether blinded to clinical info; reliability not reported	No	No: inpatients, having PET/CT to exclude other diseases for example, suspected malignancy; none had clinical evidence GCA	Yes	No (other rheumatic diseases with suspected malignancy; 11/17 RA)	Clinical diagnosis+Chuang+Healey criteria; length of follow-up not specified	Yes	Unclear	No	Yes for PMR, not stated for controls
Camellino *et al*^25^†	FDG-PET/CT	Rheumatologist and radiologist, blinded to clinical data (pers comm); reliability not reported	Yes	Little information on how patients were identified	Yes	No (65 matched controls with no inflammatory disease; 10 with treated RA)	Fulfilled Bird and ACR/EULAR criteria; median follow-up 22 months	Yes	Yes	Probably	Yes for PMR/controls, no for RA
Takahasi *et al*^24^†	FDG-PET/CT	Radiologists, blinded to clinical data [pers comm]; reliability not reported	No	No: inpatients and outpatients, having PET/CT to exclude other diseases, for example, suspected malignancy; none had clinical evidence of GCA	Yes	Maybe (untreated, elderly-onset RA)	Diagnosed by attending doctors prior to PET/CT (pers comm); diagnosis did not change on follow-up (pers comm). and verified by classification criteria	Yes	Yes	Yes	Yes

The PET or PET/CT studies that did not report data extractable into 2×2 table format are not listed here. Before-after or prognostic studies, if they did not report data extractable into 2×2 table format, are not reported here.

Incorporation bias means where the imaging (index test) informs the diagnosis (reference standard).

Diagnostic review bias means where the diagnosis (reference standard) was carried out or verified with knowledge of the imaging (index test).

*Further data were supplied by corresponding authors on request.

†Methodological details supplied by corresponding authors on request.

ACR, American College of Rheumatology; ARA, American Rheumatism Association; CTD, connective tissue disease; ESR, erythrocyte sedimentation rate; EULAR, the European League Against Rheumatism; FM, fibromyalgia; GCA, giant cell arteritis; MCP, metacarpophalangeal; OA, osteoarthritis; PET/CT, positron emission tomography CT; PMR, polymyalgia rheumatica; PsA, psoriatic arthritis; RA, rheumatoid arthritis; SpA, spondyloarthropathies.

**Table 3:** Summary data for individual tests

Anatomical finding	Studies (imaging modality)	Sensitivity (95% CI), %	Specificity (95% CI), %	Positive likelihood ratio (95% CI)	Negative likelihood ratio (95% CI)
Cervical interspinous bursitis	Salvarani *et al*^36^ (MRI)	0.83 (0.55 to 0.95)	0.69 (0.42 to 0.87)	2.7 (1.2 to 6.4)	0.24 (0.065 to 0.90)
Cervical interspinous bursitis, comparator no inflammation	Camellino *et al*^25^ (PET/CT)	0.10 (0.05 to 0.19)	0.99 (0.93 to 1.00)	13 (0.8 to 226)	0.9 (0.91 to 0.99)
Lumbar interspinous bursitis	Salvarani *et al*^37^ (MRI)	0.60 (0.31 to 0.83)	0.91 (0.62 to 0.98)	6.6 (1.0 to 46)	0.4 (0.20 to 0.96)
Lumbar interspinous bursitis, comparator no inflammation	Camellino *et al*^25^ (PET/CT)	0.46 (0.35 to 0.48)	0.99 (0.93 to 1.00)	61 (3.8 to 977)	0.54 (0.43 to 0.68)
Any interspinous bursitis	Yamashita *et al*^35^ (PET/CT)	0.79 (0.52 to 0.92)	0.82 (0.59 to 0.94)	4.5 (1.5 to 13)	0.26 (0.093 to 0.73)
Subacromial bursitis on at least one side	Cantini *et al*^33^ (USS)	0.96 (0.88 to 0.99)	0.78 (0.70 to 0.85)	4.4 (3.1 to 6.2)	0.04 (0.01 to 0.18)
	Frediani *et al*^45^ (USS)	0.70 (0.56 to 0.81)	0.61 (0.51 to 0.70)	1.8 (1.3 to 2.4)	0.49 (0.31 to 0.77)
	Falsetti *et al*^44^ (USS)	0.79 (0.62 to 0.90)	0.59 (0.42 to 0.74)	2.0 (1.2 to 3.1)	0.35 (0.16 to 0.75)
	Dasgupta *et al*^15^ (USS)	0.56 (0.47 to 0.65)	0.65 (0.58 to 0.72)	1.6 (1.2 to 2.1)	0.67 (0.53 to 0.85)
	Cantini *et al*^33^ (USS)* Frediani *et al*^45^ (USS) Falsetti *et al*^44^ (USS) Dasgupta *et al*^15^ (USS)	0.80 (0.55 to 0.93)	0.68 (0.60 to 0.75)	2.5 (1.6 to 3.8)	0.30 (0.11 to 0.81)
Subacromial bursitis on at least one side: comparator RA	Salvarani *et al*^48^ (MRI)	0.96 (0.73 to 1.00)	0.75 (0.44 to 0.92)	3.86 (1.3 to 11)	0.05 (0.003 to 0.74)
	Coari *et al*^38^ (USS)	0.09 (0.03 to 0.24)	0.90 (0.84 to 0.94)	0.95 (0.29 to 3.2)	1.01 (0.89 to 1.1)
	Dasgupta *et al*^15^ (USS)	0.56 (0.47 to 0.65)	0.72 (0.57 to 0.83)	2.0 (1.2 to 3.2)	0.61 (0.46 to 0.80)
	Ruta *et al*^39^ (USS)	0.73 (0.56 to 0.86)	0.67 (0.49 to 0.81)	2.2 (1.3 to 3.8)	0.40 (0.21 to 0.76)
Subacromial bursitis on at least one side: comparator painful shoulder conditions	Dasgupta *et al*^15^ (USS)	0.56 (0.47 to 0.65)	0.70 (0.55 to 0.81)	1.9 (1.2 to 2.9)	0.63 (0.48 to 0.83)
	Ruta *et al*^39^ (USS)	0.55 (0.43 to 0.67)	0.75 (0.63 to 0.84)	2.2 (1.3 to 3.6)	0.60 (0.44 to 0.82)
Subacromial bursitis on both sides	Cantini *et al*^33^ (USS)	0.93 (0.83 to 0.97)	0.99 (0.95 to 1.00)	106 (15 to 747)	0.07 (0.028 to 0.18)
	Frediani *et al*^45^ (USS)	0.54 (0.40 to 0.67)	0.68 (0.58 to 0.76)	1.7 (1.1 to 2.5)	0.68 (0.49 to 0.94)
	Falsetti *et al*^44^ (USS)	0.69 (0.51 to 0.83)	0.78 (0.61 to 0.89)	3.2 (1.6 to 6.3)	0.40 (0.22 to 0.70)
	Dasgupta *et al*^15^ (USS)	0.32 (0.24 to 0.41)	0.88 (0.81 to 0.92)	2.6 (1.6 to 4.2)	0.78 (0.68 to 0.89)
	Cantini *et al*^33^ (USS)* Frediani *et al*^45^ (USS) Falsetti *et al*^44^ (USS) Dasgupta *et al*^15^ (USS)	0.66 (0.36 to 0.87)	0.89 (0.66 to 0.97)	6.2 (1.2 to 32)	0.38 (0.15 to 0.97)
Subacromial bursitis on both sides: comparator RA	Dasgupta *et al*^15^ (USS)	0.32 (0.24 to 0.41)	0.78 (0.64 to 0.88)	1.5 (0.8 to 2.7)	0.87 (0.64 to 0.88)
	Ruta *et al*^39^ (USS)	0.37 (0.22 to 0.55)	0.97 (0.83 to 0.99)	11 (1.5 to 80)	0.66 (0.83 to 0.99)
Iliopsoas bursitis	Cantini *et al*^34^ (MRI)	0.50 (0.30 to 0.70)	0.80 (0.50 to 0.94)	2.5 (0.67 to 9.3)	0.63 (0.37 to 1.1)
	Cantini *et al*^34^ (USS)	0.30 (0.15 to 0.50)	0.90 (0.77 to 0.96)	3.0 (0.95 to 9.4)	0.78 (0.57 to 1.1)
Iliopectineal (iliopsoas) bursitis, comparator RA	Takahashi *et al*^24^	0.59 (0.41 to 0.75)	0.90 (0.60 to 0.98)	5.9 (0.90 to 39)	0.45 (0.28 to 0.75)
Ischiogluteal bursitis	Cantini *et al*^34^ (MRI)	0.25 (0.11 to 0.47)	0.90 (0.60 to 0.98)	2.5 (0.34 to 19)	0.83 (0.60 to 1.2)
	Yamashita *et al*^35^ (PET/CT)	0.86 (0.60 to 0.96)	0.76 (0.53 to 0.90)	3.6 (1.5 to 8.8)	0.19 (0.05 to 0.69)
	Cantini *et al*^34^ (USS)	0.20 (0.081 to 0.42)	0.95 (0.84 to 0.99)	4.0 (0.80 to 20)	0.84 (0.70 to 1.1)
Trochanteric bursitis on at least one side	Cantini *et al*^34^ (USS)	0.98 (0.81 to 1.00)	0.70 (0.54 to 0.81)	3.2 (2.0 to 5.1)	0.03 (0.002 to 0.53)
	Cantini *et al*^34^ (MRI)	0.98 (0.81 to 1.00)	0.78 (0.48 to 0.93)	4.3 (1.4 to 13)	0.031 (0.002 to 0.49)
	Dasgupta *et al*^15^ (USS)	0.21 (0.15 to 0.30)	0.91 (0.84 to 0.95)	2.3 (1.2 to 4.5)	0.87 (0.78 to 0.97)
	Yamashita *et al*^35^ (PET/CT)	0.71 (0.45 to 0.88)	0.88 (0.66 to 0.97)	6.1 (1.6 to 23)	0.32 (0.14 to 0.76)
Hand extracapsular: comparator RA	Marzo-Ortega *et al*^13^ (MRI)	0.80 (0.49 to 0.94)	0.80 (0.49 to 0.94)	4.0 (1.1 to 14)	0.25 (0.07 to 0.90)
Shoulder extracapsular: comparator RA	McGonagle *et al*^12^ (MRI)	0.64 (0.39 to 0.84)	0.86 (0.60 to 0.96)	4.5 (1.2 to 17)	0.42 (0.2 to 0.87)
Long head biceps tenosynovitis on at least one side	Cantini *et al*^33^ (USS)	0.81 (0.70 to 0.89)	0.47 (0.38 to 0.57)	1.5 (1.2 to 1.9)	0.41 (0.23 to 0.72)
	Dasgupta *et al*^15^ (USS)	0.66 (0.57 to 0.74)	0.54 (0.46 to 0.61)	1.4 (1.2 to 1.8)	0.63 (0.47 to 0.85)
	Frediani *et al*^45^ (USS)	0.68 (0.54 to 0.79)	0.59 (0.49 to 0.68)	1.7 (1.2 to 2.2)	0.54 (0.35 to 0.84)
Long head biceps tenosynovitis on at least one side: comparator RA	Coari *et al*^38^ (USS)	0.16 (0.07 to 0.32)	0.48 (0.38 to 0.58)	0.30 (0.13 to 0.69)	1.8 (1.4 to 2.3)
	Dasgupta *et al*^15^ (USS)	0.66 (0.57 to 0.74)	0.44 (0.31 to 0.59)	1.2 (0.89 to 1.6)	0.76 (0.51 to 1.2)
	Ruta *et al*^39^ (USS)	0.63 (0.46 to 0.78)	0.57 (0.39 to 0.73)	1.5 (0.89 to 2.4)	0.65 (0.37 to 1.1)
	Lange *et al*^46^ (USS)	0.14 (0.05 to 0.33)	0.59 (0.41 to 0.74)	0.33 (0.11 to 1.0)	1.5 (1.0 to 2.1)
	Coari *et al*^38^ (USS) Dasgupta *et al*^15^ (USS) Ruta *et al*^39^ (USS) Lange *et al*^46^ (USS)	0.37 (0.15 to 0.66)	0.50 (0.43 to 0.57)	0.74 (0.35 to 1.6)	1.3 (0.80 to 2.0)
	Salvarani *et al*^48^ (MRI)	0.47 (0.25 to 0.70)	0.67 (0.35 to 0.88)	1.4 (0.48 to 4.1)	0.80 (0.41 to 1.6)
Long head biceps tenosynovitis on at least one side: comparator painful shoulder conditions	Coari *et al*^38^ (USS)	0.16 (0.069 to 0.32)	0.45 (0.37 to 0.54)	0.28 (0.13 to 0.65)	1.9 (1.5 to 2.4)
	Dasgupta *et al*^15^ (USS)	0.66 (0.57 to 0.74)	0.60 (0.45 to 0.72)	1.6 (1.1 to 2.4)	0.57 (0.40 to 0.80)
	Ruta *et al*^39^ (USS)	0.47 (0.35 to 0.59)	0.80 (0.68 to 0.88)	2.3 (1.3 to 4.1)	0.67 (0.51 to 0.87)
Long head biceps tenosynovitis on both sides	Cantini *et al*^33^ (USS)	0.60 (0.47 to 0.72)	0.96 (0.90 to 0.98)	15 (5.8 to 41)	0.42 (0.30 to 0.58)
	Frediani *et al*^45^ (USS)	0.38 (0.26 to 0.52)	0.68 (0.58 to 0.76)	1.2 (0.75 to 1.9)	0.91 (0.71 to 1.2)
	Falsetti *et al*^44^ (USS)	0.62 (0.44 to 0.77)	0.66 (0.48 to 0.80)	1.8 (1.0 to 3.2)	0.58 (0.34 to 0.98)
	Dasgupta *et al*^15^ (USS)	0.37 (0.29 to 0.46)	0.73 (0.66 to 0.80)	1.4 (0.98 to 2.0)	0.86 (0.72 to 1.0)
	Cantini *et al*^33^ (USS) Frediani *et al*^45^ (USS) Falsetti *et al*^44^ (USS)* Dasgupta *et al*^15^ (USS)	0.47 (0.35 to 0.58)	0.80 (0.61 to 0.91)	2.4 (0.93 to 6.0)	0.67 (0.46 to 0.95)
Long head biceps tenosynovitis on both sides: comparator RA	Dasgupta *et al*^15^ (USS)	0.37 (0.29 to 0.46)	0.62 (0.48 to 0.75)	0.99 (0.63 to 1.5)	1.0 (0.77 to 1.3)
	Ruta *et al*^39^ (USS)	0.30 (0.17 to 0.48)	0.98 (0.86 to 1.00)	19 (1.2 to 310)	0.71 (0.56 to 0.90)
Tenosynovitis of hand extensor tendons	Cimmino *et al*^40^ (MRI)	0.67 (0.42 to 0.85)	0.69 (0.42 to 0.87)	2.2 (0.89 to 5.3)	0.48 (0.22 to 1.1)
Glenohumeral synovitis on at least one side	Cantini *et al*^33^ (USS)	0.77 (0.65 to 0.86)	0.42 (0.33 to 0.51)	1.3 (1.1 to 1.6)	0.54 (0.32 to 0.92)
	Frediani *et al*^45^ (USS)	0.66 (0.52 to 0.78)	0.65 (0.55 to 0.73)	1.9 (1.4 to 2.6)	0.52 (0.35 to 0.79)
	Falsetti *et al*^44^ (USS)	0.66 (0.47 to 0.80)	0.47 (0.31 to 0.64)	1.2 (0.81 to 1.9)	0.74 (0.40 to 1.4)
	Dasgupta *et al*^15^ (USS)	0.39 (0.30 to 0.48)	0.71 (0.64 to 0.78)	1.3 (0.96 to 1.9)	0.86 (0.72 to 1.0)
	Cantini *et al*^33^ (USS) Frediani *et al*^45^ (USS) Falsetti *et al*^44^ (USS) Dasgupta *et al*^15^ (USS)	0.62 (0.46 to 0.76)	0.58 (0.45 to 0.69)	1.5 (1.2 to 1.7)	0.66 (0.50 to 9.9)
Glenohumeral synovitis on at least one side: comparator RA	Lange *et al*^46^ (USS)	0.41 (0.23 to 0.61)	0.34 (0.20 to 0.53)	0.62 (0.35 to 1.1)	1.7 (0.93 to 3.2)
	Coari *et al*^38^ (USS)	0.66 (0.48 to 0.80)	0.52 (0.42 to 0.62)	1.4 (0.99 to 1.9)	0.66 (0.39 to 1.1)
	Ruta *et al*^39^ (USS)	0.20 (0.10 to 0.37)	0.57 (0.39 to 0.73)	0.46 (0.20 o 1.1)	1.4 (0.99 to 2.0)
	Dasgupta *et al*^15^ (USS)	0.39 (0.30 to 0.48)	0.63 (0.49 to 0.75)	1.1 (0.67 to 1.6)	0.97 (0.75 to 1.3)
	Lange *et al*^46^ (USS)† Coari *et al*^38^ (USS)† Ruta *et al*^39^ (USS) Dasgupta *et al*^15^ (USS)	0.41 (0.26 to 0.58)	0.53 (0.45 to 0.62)	0.88 (0.58 to 1.3)	1.1 (0.82 to 1.5)
	Salvarani *et al*^48^ (MRI)	0.77 (0.50 to 0.92)	0.44 (0.19 to 0.73)	1.4 (0.72 to 2.7)	0.52 (0.15 to 1.8)
Glenohumeral synovitis on at least one side: comparator painful shoulder conditions	Coari *et al*^38^ (USS)	0.66 (0.48 to 080)	0.77 (0.69 to 0.84)	2.9 (1.9 to 4.3)	0.45 (0.27 to 0.73)
	Dasgupta *et al*^15^ (USS)	0.39 (0.30 to 0.48)	0.76 (0.62 to 0.86)	1.6 (0.92 to 2.8)	0.81 (0.65 to 1.0)
	Ruta *et al*^39^ (USS)	0.12 (0.058 to 0.22)	0.93 (0.84 to 0.97)	1.8 (0.54 to 5.7)	0.95 (0.84 to 1.06)
Glenohumeral synovitis on both sides	Dasgupta *et al*^15^	0.26 (0.19 to 0.35)	0.83 (0.76 to 0.88)	1.5 (0.97 to 2.4)	0.89 (0.78 to 1.0)
	Falsetti *et al*^44^	0.48 (0.31 to 0.66)	0.66 (0.48 to 0.80)	1.4 (0.76 to 2.6)	0.79 (0.51 to 1.2)
	Frediani *et al*^45^	0.52 (0.39 to 0.65)	0.78 (0.69 to 0.85)	2.4 (1.50 to 3.7)	0.62 (0.45 to 0.84)
Glenohumeral synovitis on both sides: comparator RA	Dasgupta *et al*^15^	0.26 (0.19 to 0.35)	0.70 (0.55 to 0.81)	0.86 (0.50 to 1.5)	1.1 (0.85 to 1.3)
	Ruta *et al*^39^	0.03 (0.059 to 0.17)	0.90 (0.74 to 0.97)	0.33 (0.037 to 3.0)	1.1 (0.94 to 1.2)
Hip synovitis on at least one side	Cantini *et al*^34^ (USS)	0.45 (0.25 to 0.66)	0.55 (0.40 to 0.69)	1.0 (0.55 to 1.8)	1.0 (0.62 to 1.6)
	Frediani *et al*^45^ (USS)	0.40 (0.28 to 0.54)	0.81 (0.72 to 0.87)	2.1 (1.2 to 3.6)	0.74 (0.58 to 0.95)
	Falsetti *et al*^44^ (USS)	0.24 (0.12 to 0.42)	0.88 (0.72 to 0.95)	1.9 (0.63 to 5.9)	0.87 (0.68 to 1.1)
	Dasgupta *et al*^15^ (USS)	0.26 (0.19 to 0.36)	0.81 (0.73 to 0.87)	1.4 (0.87 to 2.3)	0.90 (0.78 to 1.0)
	Cantini *et al*^34^ (USS) Frediani *et al*^45^ (USS) Falsetti *et al*^44^ (USS) Dasgupta *et al*^15^ (USS)	0.33 (0.24 to 0.43)	0.78 (0.66 to 0.87)	1.5 (1.0 to 2.2)	0.86 (0.76 to 0.97)
	Cantini *et al*^34^ (MRI)	0.85 (0.64 to 0.95)	0.50 (0.24 to 0.76)	1.7 (0.89 to 3.2)	0.30 (0.089 to 1.0)
	Yamashita *et al*^35^ (PET/CT)	0.86 (0.60 to 0.96)	0.65 (0.41 to 0.83)	2.4 (1.2 to 4.8)	0.22 (0.058 to 0.84)
Hip synovitis on both sides	Dasgupta *et al*^15^ (USS)	0.18 (0.12 to 0.26)	0.92 (0.85 to 0.95)	2.1 (1.0 to 4.3)	0.90 (0.81 to 1.0)
	Frediani *et al*^45^ (USS)	0.32 (0.21 to 0.46)	0.83 (0.75 to 0.89)	1.88 (1.0 to 3.4)	0.82 (0.66 to 1.0)
Shoulder region uptake	Yamashita *et al*^35^ (PET/CT)	0.86 (0.60 to 0.96)	0.29 (0.13 to 0.53)	1.2 (0.84 to 1.8)	0.49 (0.11 to 2.1)
Bilateral shoulder region inflammation	Dasgupta *et al*^15^ (USS)	0.59 (0.50 to 0.68)	0.57 (0.49 to 0.65)	1.4 (1.1 to 1.7)	0.71 (0.55 to 0.92)
	Macchioni *et al*^23^ (USS)	0.45 (0.39 to 0.51)	0.60 (0.47 to 0.72)	1.1 (0.79 to 1.6)	0.91 (0.71 to 1.2)
Bilateral shoulder region inflammation: comparator painful shoulder conditions	Dasgupta *et al*^15^ (USS)	0.59 (0.50 to 0.68)	0.74 (0.61 to 0.85)	2.3 (1.4 to 3.9)	0.55 (0.42 to 0.72)
Bilateral shoulder region inflammation: comparator RA	Dasgupta *et al*^15^ (USS)	0.59 (0.50 to 0.68)	0.35 (0.23 to 0.49)	0.91 (0.70 to 1.2)	1.2 (0.75 to 1.8)
	Macchioni 2013 (USS	0.56 (0.49 to 0.63)	0.74 (0.60 to 0.85)	2.2 (1.3 to 3.7)	0.60 (0.47 to 0.76)
Hip region inflammation: comparator RA	Dasgupta *et al*^15^ (USS)	0.38 (0.30 to 0.47)	0.70 (0.55 to 0.81)	1.3 (0.77 to 2.1)	0.89 (0.70 to 1.1)
Hip region inflammation: comparator painful shoulder conditions	Dasgupta *et al*^15^ (USS)	0.38 (0.30 to 0.47)	0.83 (0.70 to 0.91)	2.3 (1.2 to 4.4)	0.74 (0.61 to 0.90)
One shoulder and one hip region inflammation	Dasgupta *et al*^15^ (USS)	0.33 (0.26 to 0.42)	0.84 (0.77 to 0.89)	2.1 (1.3 to 3.2)	0.80 (0.69 to 0.92)
	Macchioni *et al*^23^ (USS)	0.34 (0.27 to 0.41)	0.77 (0.68 to 0.85)	1.5 (0.97 to 2.3)	0.86 (0.74 to 1.0)

Note these are reported to 2 significant figures but note CIs are often wide. Sensitivity, specificity, positive likelihood ratio and negative likelihood ratio are given for individual tests or, where possible, a summary value is calculated by meta-analysis. Sensitivity and specificity are given to two decimal places. Likelihood ratios are given to two significant figures unless <0.1 or >10.

*Outlier by visual inspection of HsROC.

†Influential outlier (Cooks’ distance >3). “Shoulder region inflammation”, “hip region inflammation” are defined as per ACR/EULAR provisional classification criteria for PMR.^15^

ACR, American College of Rheumatology; EULAR, the European League Against Rheumatism; HsROC, Hierarchial Summary Receiver-Operator Characteristic Curve; PMR, polymyalgia rheumatica.

### Accuracy of bursitis imaging (extracapsular inflammation)

**Figure 2:**
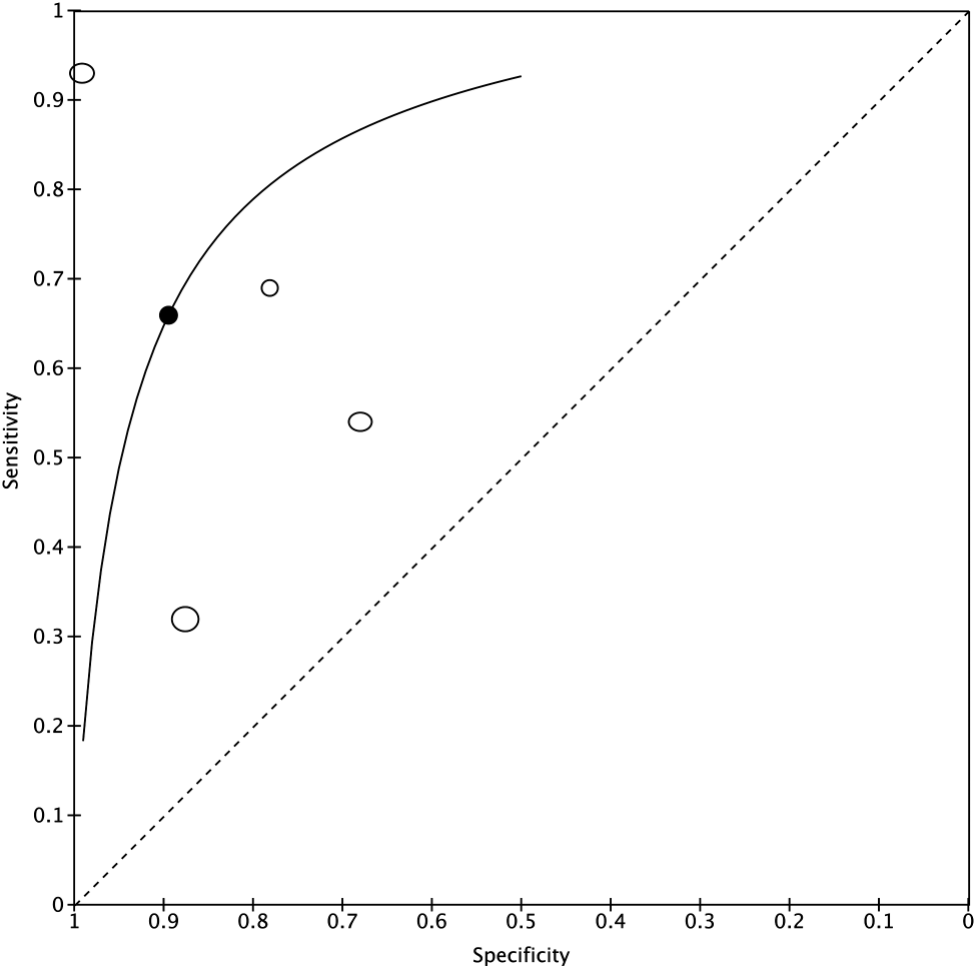
Summary ROC plot for bilateral subacromial bursitis. ROC, receiver operating characteristic.

Meta-analysis of four USS studies gave a sensitivity of 80% (95% CI 55% to 93%) and specificity of 68% (60% to 75%) for SAB; the same studies showed a sensitivity of 66% (36% to 87%) and specificity 89% (66% to 97%) for bilateral SAB. Examination of the HsROC plot indicates substantial heterogeneity of discrimination, with an early study^33^ showing much higher diagnostic accuracy than subsequent studies (figure 2).

Data on trochanteric bursitis were variable; very high sensitivity of ultrasound in an early single-centre study^34^ was not replicated in a later multicentre study.^15^ Pelvic-girdle symptoms were required for inclusion in the earlier study, whereas the later study required shoulder symptoms but did not require pelvic-girdle symptoms.

Other bursal sites around the hip/pelvic region (ischiogluteal, iliopsoas), while reportedly more specific for PMR than trochanteric bursitis, are technically difficult to detect using ultrasound compared to MRI.^34^ Although a PET/CT study suggested inflammation around the ischial tuberosity may be informative for PMR diagnosis, the sample size was small, and thus CIs for sensitivity and specificity are wide;^35^ sensitivity on an earlier MRI study was only 25%.^34^ Similarly, inflammation (bursitis) between posterior vertebral elements, detectable by PET/CT^25^ ^35^ or MRI,^36^ ^37^ appeared to be highly specific compared to age-matched controls without inflammatory rheumatic disease, but may also be observed in RA;^25^ most of the RA comparator patients were taking prednisolone (D Camellino, personal communication, January 2015). PET/CT can also identify iliopsoas (iliopectineal) bursitis, sometimes seen in RA as well.^24^

### Accuracy of imaging intracapsular inflammation and fluid around long head of biceps tendon

Synovitis at shoulder (glenohumeral) or hip (coxofemoral) joints, and fluid around the long head of biceps tendon (which is related to synovial inflammation, since this space is synovium-lined and also communicates with the glenohumeral joint itself), were reported by several studies. Combining the ultrasound studies, glenohumeral synovitis had a sensitivity of 62% (95% CI 46% to 76%) and specificity of 58% (45% to 69%), and hip synovitis had a sensitivity of 33% (24% to 43%) and specificity of 78% (66% to 87%). MRI and PET/CT were much more sensitive for detecting hip synovitis in PMR, but with a loss of specificity.

### Comparison with RA

Comparison with RA may identify imaging features specific to PMR and not seen in other inflammatory joint diseases. Two ultrasound studies recruited only patients with RA as controls. In both studies, to minimise the risk of misclassification, cases and controls were selected on the basis of already having an established diagnosis of (treated) PMR or RA. Methodological quality was difficult to assess in the earlier study,^38^ but the later study^39^ recruited only relapsing patients with new-onset bilateral shoulder pain; the authors reported in correspondence with us that low-dose prednisolone treatment did not seem to affect the ultrasound findings. It is difficult to recruit large numbers of patients with untreated RA and elevated inflammatory markers. One PET/CT study recruited 10 untreated RA patients^24^ but in another, the RA patients were on treatment.^40^

### Combined features (defined by the provisional ACR/EULAR classification criteria for PMR

In the provisional ACR/EULAR classification criteria for PMR,^15^ ultrasound features of inflammation were combined. First, different anatomical sites from each region were combined: *shoulder region inflammation* was defined as SAB, fluid around the long head of biceps tendon, OR glenohumeral synovitis; *hip region inflammation* was defined as coxofemoral synovitis OR trochanteric bursitis.^15^ This use of OR had the effect of increasing sensitivity of the criteria. Second, based on the regression modelling used to define the final classification criteria set, one point was allocated for bilateral shoulder region inflammation, and one point for shoulder region inflammation plus hip region inflammation. This requirement for two regions involved had the effect of increasing specificity of the criteria. Bilateral shoulder region involvement had a sensitivity of 59% (50% to 68%) and specificity 57% (49% to 65%), whereas having one shoulder and one hip involved had a sensitivity 33% (26% to 42%) and specificity 84% (77% to 89%).^15^ This may reflect the requirement for shoulder symptoms for inclusion of both patients and controls, whereas PMR characteristically causes symptoms at shoulders as well as hips. A later study produced similar sensitivity/specificity data, with the caveat that its control population was patients with early RA, and the completeness of sonographer and diagnostician blinding to each other's findings was unclear.^23^

### Change with treatment

Comparison of before-treatment and after-treatment findings was reported for musculoskeletal USS,^28–30^ MRI^31^ and FDG-PET.^41^ After 4 weeks of glucocorticoid treatment, shoulder USS normalised in half of the patients who had had bilateral USS abnormalities before treatment, and this persisted to 6 months.^28^ In a second study, 11/24 patients had power Doppler signal (indicating microvascular hyperaemia, and suggesting chronicity of inflammation) in at least one shoulder structure; this was present in only 1/24 patients at 6 months. PMR still had abnormalities in shoulder ultrasound at 6 months compared to a group of 21 ‘normal’ patients, but this was not seen for hip ultrasound findings.^30^

### Prognosis

In 57 patients with PMR, the presence of power Doppler signal prior to treatment in articular/periarticular shoulder structures significantly predicted PMR relapse/recurrence after 6 months.^29^

### Direct comparison of test accuracy using paired data

Only two couples of tests had sufficient paired data (≥4 studies) for our analysis. The paired comparisons were only made where the relevant tests were carried out on cases and controls in all studies, so the same cases and controls had both tests. Ultrasound detection of bilateral SAB was compared to ultrasound detection of hip synovitis, but the two sROC's had different shapes and comparison of overall accuracy was not possible. Ultrasound detection of subacaromial-subdeltoid bursitis was compared to ultrasound detection of glenohumeral synovitis. The two sROC's had similar shapes, and we found ultrasound detection of SAB to be significantly more accurate than ultrasound detection of glenohumeral synovitis, for the diagnosis of PMR (p=0.004).

## Discussion

Our objective was to determine whether musculoskeletal imaging is accurate enough to be useful to support clinical diagnosis of PMR. Although MRI and PET/CT revealed potentially characteristic features of focal inflammation between vertebral processes and within the pelvis, only the USS studies had enough control patients with inflammatory diseases for precise estimates of diagnostic accuracy. The most informative single USS feature appeared to be bilateral SAB, with a specificity of 89% (95% CI 66% to 97%) and sensitivity 66% (43% to 87%); however, the earliest study reported much higher diagnostic accuracy than subsequent studies. In general, substantial clinical and statistical between-study heterogeneity was noted, including important biases, and therefore the absolute sensitivity/specificity estimates given here must be interpreted with great caution. The effects of between-study heterogeneity can be minimised where each study reported the same two tests; this pairwise comparison across four studies showed that SAB was significantly more accurate than glenohumeral synovitis for PMR diagnosis. This suggests that it might not be appropriate to weight these two features equally in diagnosing PMR, as has been suggested by the latest criteria set.^15^

Several potential biases were identified during the quality assessment. First, all studies, except one, had a case-control study design. This would introduce spectrum bias and produce heterogeneity in the specificity estimates depending on how the controls were recruited. The only study with a diagnostic cohort design suffered from incorporation bias because the ultrasound was used to help make the diagnosis. Second, most of the reports contained little detail on how blinding was achieved and maintained. This is particularly difficult for USS, which requires close patient contact. Recruitment of two controls following each case could have compromised blinding and was associated with much higher estimates of diagnostic accuracy.^33^ ^34^ Blinding of the treating rheumatologist and the patient until after the final adjudication of reference-standard clinical diagnosis (which may be 1 year later), would require explicit patient consent and may not always have been possible. There was often insufficient detail on whether and how the patients themselves were blinded to their imaging findings, and on how frequently the treating clinician had to be unblinded or patients excluded from analysis because of unexpected findings on the scan (particularly relevant for MRI and PET studies). Lastly, intra/inter-rater reliability of imaging test was rarely fully reported, although this was arguably unlikely to introduce a systematic bias.

Some limitations of this analysis could have made imaging appear less accurate than it really is. First, the use of binary scores (present/absent) rather than grades of intensity of inflammation or number of sites involved is a limitation of diagnostic accuracy meta-analysis methods. Second, the necessity of using rheumatologist diagnosis as an (imperfect) reference standard; for future studies, adding a ‘test of treatment’^42^ might be used to improve the reference standard, since ultrasound abnormalities were associated with complete response to glucocorticoid therapy.^30^ Third, it is not known whether adding power Doppler to the ultrasound might offer superior diagnostic accuracy for PMR compared to grey-scale ultrasound alone.

Overall, the accuracy of musculoskeletal imaging tests cannot currently be accurately quantified for clinical diagnosis of PMR, primarily due to the limited amount of published data and biases in the studies. The reference standard is still rheumatologist diagnosis, which may use clinical intuition rather than formal criteria;^43^ we might expect that tests adding additional information, including imaging tests, might help in ‘grey cases’ where the clinical diagnosis is not clear-cut, but there are no studies recruiting these ‘grey cases’ and evaluating them without incorporation bias. Finally, if the prognostic value of imaging were known, this might also have value for clinical practice and perhaps even for patient classification. This type of evidence would help determine the optimal place of imaging tests in diagnostic care pathways for patients with suspected PMR.
